# Non-Linear Concentration-Response Relationships between Ambient Ozone and Daily Mortality

**DOI:** 10.1371/journal.pone.0129423

**Published:** 2015-06-15

**Authors:** Sanghyuk Bae, Youn-Hee Lim, Saori Kashima, Takashi Yorifuji, Yasushi Honda, Ho Kim, Yun-Chul Hong

**Affiliations:** 1 Public Health Medical Service, Seoul National University Hospital, Seoul, Korea; 2 Environmental Health Center, College of Medicine, Seoul National University, Seoul, Korea; 3 Department of Public Health and Health Policy, Institute of Biomedical & Health Science, Hiroshima University, Hiroshima, Japan; 4 Department of Human Ecology, Okayama University Graduate School of Environmental and Life Science, Okayama, Japan; 5 Department of Health Care Policy and Health Economics, Faculty of Medicine, University of Tsukuba, Tsukuba, Japan; 6 Department of Epidemiology and Biostatistics, Graduate School of Public Health, Seoul National University, Seoul, Korea; 7 Department of Preventive Medicine, College of Medicine, Seoul National University, Seoul, Korea; 8 Institute of Environmental Medicine, Seoul National University Medical Research Center, Seoul, Korea; Institute for Health & the Environment, UNITED STATES

## Abstract

**Background:**

Ambient ozone (O_3_) concentration has been reported to be significantly associated with mortality. However, linearity of the relationships and the presence of a threshold has been controversial.

**Objectives:**

The aim of the present study was to examine the concentration-response relationship and threshold of the association between ambient O_3_ concentration and non-accidental mortality in 13 Japanese and Korean cities from 2000 to 2009.

**Methods:**

We selected Japanese and Korean cities which have population of over 1 million. We constructed Poisson regression models adjusting daily mean temperature, daily mean PM_10_, humidity, time trend, season, year, day of the week, holidays and yearly population. The association between O_3_ concentration and mortality was examined using linear, spline and linear-threshold models. The thresholds were estimated for each city, by constructing linear-threshold models. We also examined the city-combined association using a generalized additive mixed model.

**Results:**

The mean O_3_ concentration did not differ greatly between Korea and Japan, which were 26.2 ppb and 24.2 ppb, respectively. Seven out of 13 cities showed better fits for the spline model compared with the linear model, supporting a non-linear relationships between O_3_ concentration and mortality. All of the 7 cities showed J or U shaped associations suggesting the existence of thresholds. The range of city-specific thresholds was from 11 to 34 ppb. The city-combined analysis also showed a non-linear association with a threshold around 30-40 ppb.

**Conclusion:**

We have observed non-linear concentration-response relationship with thresholds between daily mean ambient O_3_ concentration and daily number of non-accidental death in Japanese and Korean cities.

## Introduction

Ozone (O_3_), a component of the troposphere, is formed by photochemical reaction between precursor chemicals, such as volatile organic carbon (VOC) and oxides of nitrogen (NO_x_), which are emitted from both anthropogenic and natural sources. In nature, VOCs are emitted from vegetation, and NO_x_ from wild fire or soil [1]. The O_3_ has existed in the troposphere at the level of background concentration (20–45 ppb) due to the natural sources of precursor chemicals. However, the ambient concentration of O_3_ has been elevated since the beginning of the 20^th^ century due to the increased anthropogenic emission caused by industrialization and increased use of motor vehicles [2].

O_3_ in the stratosphere blocks harmful ultraviolet rays, and benefits human health. However, the high concentration of tropospheric O_3_ increases airway resistance and decreases lung function when inhaled. Consequently, exposure to ambient O_3_ has been reported to exacerbate respiratory diseases and increase the risk of death [3–6]. The exposure to ambient O_3_ also causes inflammation and oxidative stress in lungs, the putative mechanism of increased risk of cardiovascular morbidity and mortality [7–10].

Association between ambient O_3_ concentration and mortality has been reported since the 1990s, but inconsistent results from time-series analyses have been reported in single-city studies [11]. For instance, an analyses conducted in Melbourne, Australia in 2000 showed that the daily number of death increased by 0.11% (95% CI: 0.03–0.19%) for the increment of 1 μg/m^3^ [12], while another study conducted in Incheon, Korea in 1999 reported a statistically significant negative association [13]. However, multi-city studies and meta-analyses have consistently reported that increased O_3_ exposure was associated with increased mortality [11,14,15].

There has also been inconsistent reports regarding the shape of concentration-response (C-R) relationship and existence of threshold. Previous studies have suggested the possibility of a non-linear C-R association [9,16–18]. On the other hand, Bell et al. reported that they could not find evidence supporting the threshold [19] in a study analyzing National Morbidity, Mortality, and Air Pollution Study (NMMAPS) dataset. Another study conducted in United Kingdom analyzed data from 10 regions and reported that only the largest city, London, had a threshold in the association between ambient O_3_ concentration and daily mortality [20]. Atkinson et al. had suggested that lack of power likely contributed to non-detection of threshold in other places than London [20].

The shape of C-R relationship has important public health implications. If the relationship is not linear and has a threshold, it means that no adverse health effect exists below the threshold, and this should be reflected in establishing the air quality standard. The reports from agencies that establish air quality standard acknowledge the possibility of non-linear C-R relationship and existence of threshold, but it has been inconclusive since not enough studies on the matter have been conducted [1,21].

In light of securing power, metropolitan cities in Japan and Korea should be good places to analyze the C-R relationship between ambient O_3_ concentration and mortality. Also, conducting a multi-city study in two countries would provide more comprehensive evidence, since, to the best of our knowledge, only one single-city study has been conducted to examine C-R relationship of O_3_ and mortality in Asia. Therefore, we aimed to examine the C-R relationship between ambient O_3_ concentration and daily mortality in 13 metropolitan cities in Japan and Korea, and explored the possibility of the presence of a threshold.

## Methods

### Study population

In a previous study, power was one of the speculated reason of not detecting the threshold [20], therefore we selected 13 metropolitan cities with population over 1 million in 2000 to secure power. The selected cities were Sapporo, Tokyo, Nagoya, Osaka, Kitakyushu and Fukuoka in Japan, and Seoul, Incheon, Daejon, Daegu, Gwangju, Busan and Ulsan in Korea. Sendai, Japan also had a population over 1 million in 2000, but the data for ambient O_3_ concentration was not available.

### Data

Daily mean ambient concentration of O_3_ from Jan. 1, 2000 to Dec. 31, 2009 was calculated from hourly measurement of background monitoring stations in each city operated by the Ministry of Environment of Japan and the Ministry of Environment of Korea. The data was provided by Seoul Research Institute of Public Health and Environment of Korea (http://health.seoul.go.kr/life_health) and National Institute for Environmental Studies of Japan (https://www.nies.go.jp/gaiyo/index-e.html). The measured concentrations from monitors in each city were averaged to estimate the city-wide concentration. Daily mean concentration of particulate matter with aerodynamic diameter less than 10 μm (PM_10_) was calculated from the hourly measurement from the same monitoring stations that measured O_3_ in Korea. Since suspended particulate matter (SPM), instead of PM_10_, was measured in the monitoring stations that measured O_3_ in Japan, we converted SPM to PM_10_ using the conversion factor (PM_10_ = SPM ×1.16) provided by the Ministry of Environment of Japan.

Daily number of deaths from non-accidental causes (ICD-10 code: A00-T98) at the age of ≥30 years in the study period, were extracted from vital statistics of both countries, which were kept by the Statistics Bureau, Ministry of Internal Affairs and Communications of Japan (http://www.stat.go.jp/english/index.htm) and the Statistics Korea (http://kosis.kr/eng/). We also extracted the daily number of deaths from respiratory (ICD-10 code: J00-J99) and cardiovascular diseases (ICD-10 code: I10-I70).

The weather variables including daily mean temperature (°C) and relative humidity (%) were extracted from databases of the Japan Meteorological Agency (http://www.jma.go.jp/jma/indexe.html) and the Korea Meteorological Administration (http://web.kma.go.kr/eng/). The data for each city were available for Korean cities. However, relevant prefectural data were used for Japanese cities because of the limitation of data availability.

The yearly population of each city were extracted from Statistics Japan [22] and Korean Statistical Information Service [23], respectively.

### Statistical Analysis

First, we conducted city-specific analyses. Generalized additive models (GAM) were constructed for each city to examine the non-linearity of the association between O_3_ concentration and daily number of deaths. We constructed two separate models, one with cubic spline for O_3_ (non-linear model) and another with linear O_3_ term (linear model) for each city. For the spline model we applied degrees of freedom of 3 for O_3_. Daily mean temperature and daily mean relative humidity were both included in the model with cubic splines. Daily mean PM_10_ concentration, day of the week, holidays, season and time trend were also included as covariates in the model. Time trend was included in the model as natural cubic spline of calendar date with degree of freedom of 2 per year, and seasonality was controlled by including indicator variable for seasons (warm: April through September, cold: October through March) and year. To determine appropriate lag of O_3_ exposure, we analyzed for each and moving average from lag0 to lag5. Various lags of temperature (lag 0, moving averages of lags 0–1, 0–3, 0–7, 0–14 and 0–28) were also explored [24]. The yearly population of each city was also included as an offset, since the population of the cities had changed during the study period. For instance, the population of Ulsan, Korea had increased by 134,171 (25%) from 2000 to 2009.

We compared the Akaike Information Criterion (AIC) of the non-linear model with the linear model. The AIC is a likelihood-based model selection statistic with lower values indicating a better fit of the underlying data: AIC = 2k – 2ln(L), where k is the number of parameters in the statistical model and L is the maximized value of the likelihood function for the estimated model [25]. We used ‘AIC’ function of ‘mgcv’ package for R to calculate AIC of each model. We calculated the difference of AIC between linear and non-linear models (ΔAIC = AIC of spline model—AIC of linear model). We also analyzed the difference of the deviances of the models, and the statistical significance was tested [26]. The shape of the plot of the spline models were also examined.

We considered cities to have non-linear C-R relationship with the possibility of threshold when all the following three criteria were met; 1) ΔAIC was negative, suggesting better fit of non-linear model, 2) the difference of the deviance was significant, and 3) the curve was J or U shaped. PM_10_ concentration was further adjusted in additional models.

We also constructed models to analyze the association of O_3_ concentration with the respiratory and cardiovascular mortality. As a sensitivity analysis, we constructed another model using daily maximum 1-hour concentration of O_3_ as independent variable for the cities in Korea. We could not perform same sensitivity analysis in the Japanese cities because the maximum 1-hour concentration was not available. We used ‘mgcv’ package[27] with R version 3.0.2 (R Foundation for Statistical Computing, Vienna, Austria) for the city-specific analyses.

After the city-specific analyses, city-combined analyses were conducted. We pooled all city-specific data and constructed generalized additive mixed model (GAMM) to account for the between- and within-city variances by applying random effect for an indicator variable for city in the model. The same covariates as the city-specific models were also included in the city-combined model. The linearity of the association was examined again by comparing the AIC between linear and non-linear models and examining the shape of the plot. The ‘gamm4’ package[28] for R was used for the city-combined analyses.

For the cities which met the criteria of non-linear C-R relationship in city-specific analyses, we explored the thresholds by conducting piecewise linear regression analyses. We used the ‘HEAT’ package [29] for R to conduct the analyses. We constructed linear-threshold models for each 1 ppb of O_3_ concentration in the range of 10–60 ppb to search for thresholds, and compared the AICs of each model. The O_3_ concentration that showed the smallest AIC was considered to be the threshold. To estimate the 95% confidence interval (CI) of the thresholds, we used bootstrap procedure with 5,000 repetition of linear-threshold model analyses for each city in the range of ±10 ppb from the original estimate. Each bootstrap cycle randomly selected the same number of observations as the original dataset with replacement, and estimated the threshold. We excluded those cycles that failed to converge, and then we estimated 95% CI from the distribution of estimated thresholds using percentile method. We used package ‘boot’ for R [30].

Scatter plots were used to examine the possible relationship between the threshold and city-specific characteristics including mean temperature, mean O_3_ concentration and mean PM_10_ concentration.

## Results

Table 1 shows the characteristics of the cities. Tokyo had the largest population of age ≥ 30 (7,960,170) and Ulsan had the smallest (529,991). Since we analyzed for the number of death at the age of ≥ 30 years, the study population of the 3 smallest cities (Ulsan, Daejon, Gwangju) were less than 1 million. Busan had the highest mean O_3_ concentration and Seoul had the lowest. The mean O_3_ concentration did not differ greatly between Korea and Japan, which were 26.2 ppb and 24.2 ppb, respectively. However, the mean daily PM_10_ concentration was generally lower in Japanese cities compared to Korean ones. The mean temperature was highest in Kitakyushu and Fukuoka, and lowest in Sapporo. The 75^th^ percentile of daily mean O_3_ concentration did not exceed 40 ppb in all cities.

**Table 1:** Characteristics of study cities in Japan and Korea.

City	Population in 2000 (age ≥ 30)	Area (km^2^)	No. of O_3_ monitors	O_3_ measurement interval	O_3_ measurement method method	Mean O_3_ (ppb)	Mean PM_10_ (μg/m^3^)^*^	Mean Temp. (°C)^†^	Mean Humidity (%)^†^	Mean no. of daily death
Korea										
Seoul	5,539,201	605.50	25	continuous	UV absorption	22.0±12.6	64.2±45.4	12.9±10.1	61.6±14.6	100.4±11.9
Incheon	1,347,005	964.53	15	continuous	UV absorption	25.8±12.1	59.5±34.9	12.7±9.6	67.2±14.4	28.8±5.8
Daejon	676,883	539.83	8	continuous	UV absorption	26.1±12.9	48.0±32.3	13.1±9.7	65.6±13.7	15.2±4.1
Daegu	1,334,839	885.60	11	continuous	UV absorption	27.7±13.3	58.6±33.9	14.6±9.3	57.6±16.6	30.0±6.1
Gwangju	705,804	501.44	7	continuous	UV absorption	25.7±11.9	51.0±32.2	14.1±9.2	66.4±16.1	15.4±4.1
Busan	2,077,738	759.86	19	continuous	UV absorption	29.4±11.1	57.8±33.3	14.8±7.9	62.8±18.7	49.3±7.9
Ulsan	529,991	1,056.29	14	continuous	UV absorption	27.0±10.6	50.7±33.0	14.6±8.4	61.1±17.7	10.5±3.3
Japan										
Sapporo	1,816,597	1,121.12	9	continuous	UV absorption	24.2±10.6	14.4±9.5	9.2±9.4	68.3±10.8	34.9±7.2
Tokyo	7,960,170	621.35	24	continuous	UV absorption	22.7±12.2	35.7±18.9	16.7±7.7	59.3±15.4	171.6±22.8
Nagoya	2,132,762	326.45	13	continuous	UV absorption	22.2±11.5	40.0±19.6	16.2±8.5	64.4±12.1	45.1±8.9
Osaka	2,501,961	222.11	14	continuous	UV absorption	25.2±12.3	36.6±19.3	17.2±8.3	32.8±10.5	68.9±15.6
Kitakyushu	1,002,909	487.66	14	continuous	UV absorption	28.7±12.8	31.8±18.3	17.4±7.8	65.1±10.9	24.7±5.9
Fukuoka	1,329,832	340.60	8	continuous	UV absorption	28.4±12.2	35.9±18.7	17.4±7.8	65.1±10.9	22.3±5.5

* SPM was converted with conversion factor of 1.16 to PM10 for Japanese cities.

† Relevant prefecture data was used for Japanese cities.

We selected lag0-1 for further analyses because it showed the greatest ΔAIC between non-linear and linear models in most of the cities (Table A in S1 File). We also analyzed for various lags of daily mean temperature. Although the different temperature lags did not change the patterns of non-linearity, the greatest ΔAIC was observed for lag0 (Table B in S1 File). Hence, we used lag0 of temperature in the subsequent analyses. Table 2 shows the associations of O_3_ concentration and daily number of deaths and the comparison of the AIC between linear and spline models. Ten out of 13 cities had negative ΔAIC suggesting a better fit for spline models, and 7 among them had significant difference of deviance between linear and spline models. All of the 7 cities showed J or U shaped associations suggesting existence of a threshold (Fig A in S1 File). Those 7 cities were Seoul, Daegu, Busan, Sapporo, Tokyo, Nagoya and Kitakyushu. The associations between O_3_ concentration and cardiovascular mortality showed similar results, especially in Seoul and Tokyo. However, the respiratory mortality did not show non-linear association (Table C in S1 File). The models using daily maximum 1-hour concentration of O_3_ showed a similar pattern. The negative ΔAIC and J or U shaped associations were observed in Seoul, Daejon, Daegu and Busan (Table D and Fig B in S1 File)

**Table 2:** Associations between daily mean O_3_ (lag_0-1_, ppb) and daily number of mortality adjusted for PM_10_ in 13 Japanese and Korean cities from 2000 to 2009

City	Linear Model				Spline Model		Comparison of models	
	Beta	SE	P-value	AIC	P-value	AIC	ΔAIC	P-value^*^
Korea								
Seoul	-0.00024	0.000247	0.3412	27824.3	0.0002	27808.0	-16.3	<0.0001
Incheon	-0.00065	0.000418	0.1199	22731.3	0.2592	22731.0	-0.3	0.1121
Daejon	-0.00030	0.000575	0.6046	20058.0	0.6046	20058.0	0.0	0.0016
Daegu	0.00036	0.000421	0.3916	22885.3	0.0588	22881.4	-3.9	0.0125
Gwangju	0.00094	0.000618	0.1300	20248.8	0.1301	20248.8	0.0	0.0039
Busan	0.00095	0.000330	0.0039	24719.2	0.0000	24709.1	-10.1	0.0004
Japan								
Ulsan	0.00101	0.000708	0.1537	18690.8	0.1537	18690.8	0.0	0.0026
Sapporo	-0.00014	0.000439	0.7481	23314.4	0.1761	23311.7	-2.7	0.0286
Tokyo	-0.00081	0.000173	0.0000	30326.9	0.0000	30290.8	-36.1	<0.0001
Nagoya	-0.00056	0.000380	0.1400	24704.7	0.0011	24693.7	-11.0	0.0002
Osaka	-0.00124	0.000281	0.0000	26212.4	0.0001	26210.8	-1.6	0.0504
Kitakyushu	-0.00091	0.000403	0.0241	22300.2	0.0155	22296.4	-3.7	0.0128
Fukuoka	-0.00054	0.000475	0.2530	21987.5	0.2460	21987.4	-0.1	0.1373

ΔAIC = AIC of Spline model–AIC of Linear model

*For the difference of the deviances between the linear and spline models.

**Fig 1:**
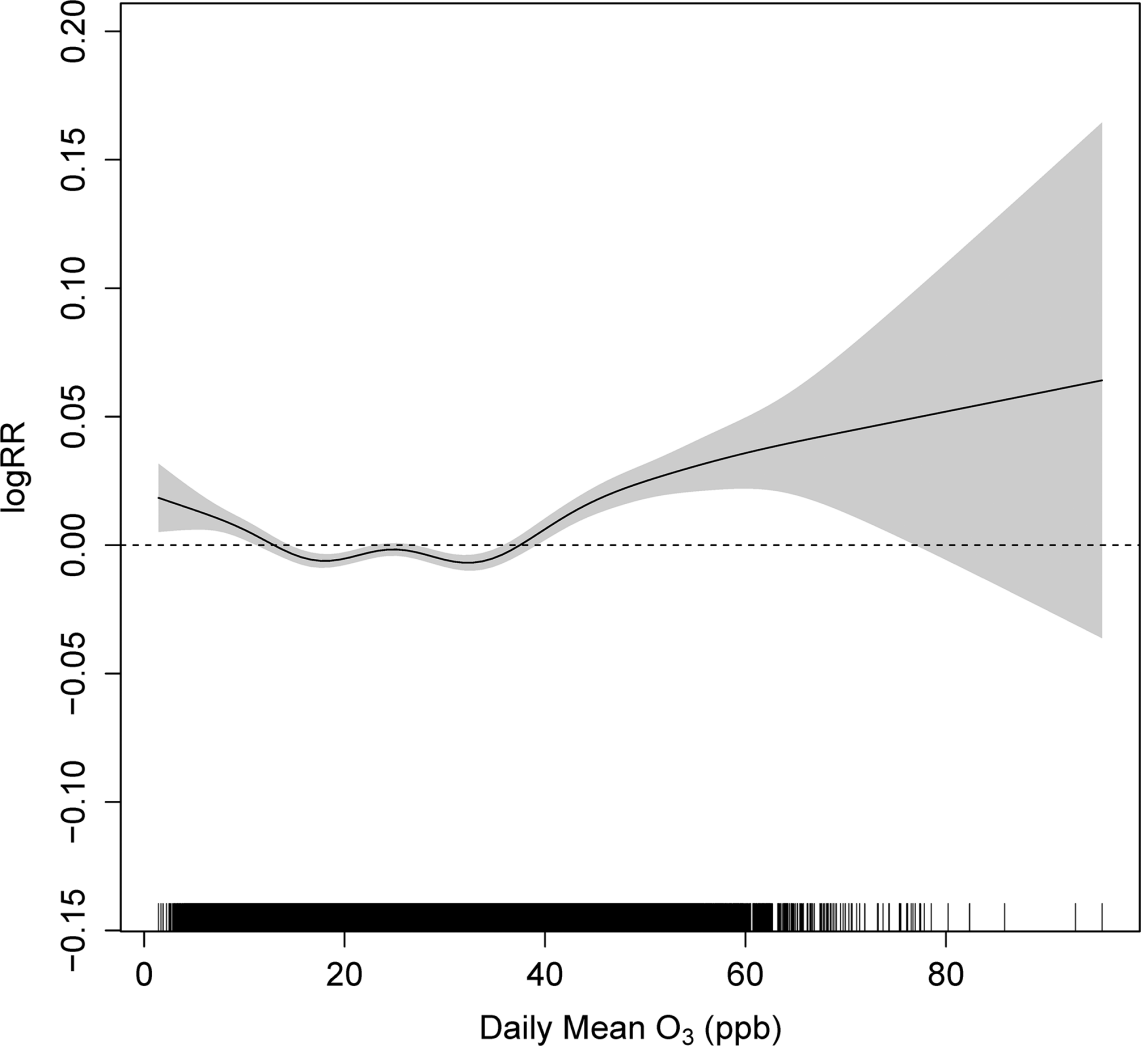
City-combined association between daily mean O_3_ (lag0-1, ppb) and daily mortality adjusted for PM_10_ in 13 Japanese and Korean cities from 2000 to 2009.

The city combined analyses of all 13 cities also showed that the AIC of spline model was smaller than that of linear model (ΔAIC = -101.3). The shape of the plot was also J-shaped, suggesting a threshold between 30–40 ppb (Fig 1).

For the 7 cities with a non-linear C-R relationship, the thresholds determined by piecewise linear regression analyses varied from city to city with a range of 11–34 ppb (Table 3). The association over the threshold were statistically significant in Daegu, Busan, Tokyo and Kitakyushu, and the excessive risk of mortality for the 1 ppb increment of O_3_ above the thresholds were 0.08%, 0.12%, 0.17% and 0.17%, respectively. The associations below the thresholds were significant in Seoul, Daegu, Busan, Tokyo and Nagoya, and the excessive risk of mortality for the 1 ppb increment of O_3_ below the thresholds were -0.43%, -0.32%, -0.46%, -0.12%, and -0.18%, respectively. The thresholds seem to be lower with low mean temperature (Fig C in S1 File).

**Table 3:** Thresholds of concentration-response relationship between O_3_ concentration and daily mortality and the excessive mortality for 1 ppb increment below and above the thresholds adjusting for PM_10_ in 7 Japanese and Korean cities.

City	Threshold [ppb, (95% CI)]	Excessive mortality [Below the threshold, % (95% CI)]	Excessive mortality [Above the threshold, % (95% CI)]
Seoul	15 (12–17)	-0.43(-0.57,-0.29)	0.02(-0.02,0.06)
Daegu	18 (13–23)	-0.32(-0.60,-0.05)	0.08(0.00,0.16)
Busan	21 (15–31)	-0.46(-0.68,-0.24)	0.12(0.06,0.18)
Sapporo	11 (8–19)	-1.14(-2.35,0.07)	0.00(-0.06,0.06)
Tokyo	34 (29–40)	-0.12(-0.16,-0.08)	0.17(0.09,0.25)
Nagoya	22 (19–37)	-0.18(-0.30,-0.06)	0.06(-0.04,0.16)
Kitakyushu	32 (26–41)	-0.08(-0.20,0.04)	0.17(0.05,0.29)

## Discussion

We examined the linearity of C-R relationships between O_3_ concentration and mortality in 13 metropolitan cities in Japan and Korea, from 2000 to 2009. We also explored the thresholds in the cities with non-linear C-R relationships. We observed that 7 out of 13 cities had a non-linear C-R relationship, and all of them (Seoul, Daegu, Busan, Sapporo, Tokyo, Nagoya and Kitakyushu) had thresholds. The non-linearity did not diminish even after adjustment for PM_10_ concentration. The thresholds varied in the 7 cities, and seemed to be lower with lower mean temperature. The city-combined analysis also showed a non-linear C-R relationship with a threshold at 30–40 ppb, being robust with adjustment for PM_10_ concentration.

O_3_ has existed at background level due to natural emission of precursor chemicals, and speculatively has no adverse health effect below the background concentration [1,17]. Experimental studies at the individual level have also suggested the existence of threshold. In a controlled exposure study with volunteers, the association between O_3_ concentration and parameters of lung function test were reported to be non-linear [31,32]. The mechanism of health effects from O_3_ exposure also suggest a threshold. It is considered that inflammation and oxidative stress, along with direct irritation, causes the health effect [33], and these effects should present only after the level of exposure exceeds the capacity of physiological defense mechanisms. Since defensive capacity varies between individuals and this variation would mask the threshold, it was thought that the threshold at the population level would be difficult to observe [1,21].

Earlier studies to elucidate the threshold of the association between O_3_ concentration and mortality have been conducted without definitive conclusion. For instance, 3 studies analyzing NMMAPS dataset reported contradicting results. Bell et al. analyzed the NMMAPS dataset to explore the threshold. However, they reported that they could not find the evidence for a threshold, and concluded that if it existed, it would be lower than the current air quality standard [19]. Later, Smith et al. also analyzed the NMMAPS dataset and reported that the effect of O_3_ exposure on mortality was not homogeneous at different levels of exposure[17]. Another recent study, also on analyzing the NMMAPS dataset, reported that the association between O_3_ and mortality was non-linear and the threshold was observed around 30 ppb [18]. However, the O_3_ concentration observed in these previous studies were relatively higher, making it difficult to examine the effect at a lower concentration. The O_3_ concentration in the present study was relatively lower than that of the NMMAPS dataset (for instance, the proportion of days with daily mean O_3_ concentration ≤ 20 ppb was 27% in the NMMAPS dataset [19], while it was 34% in the present study) and this may have increased the probability of detecting the threshold.

We observed different thresholds among the cities. A previous study analyzing the data from 9 cities in United States reported that 6 cities showed non-linear C-R relationship and different thresholds ranging 10–45 ppb [34]. However, little is known on why cities have different thresholds. In the present study, the thresholds seemed to be associated with mean temperature and possibly with PM_10_ concentration. Since an additive interaction has been reported for O_3_ and PM_10_ [35], and the association of O_3_ with mortality attenuated by 20–30% when the association was adjusted for PM_10_ [17], it is possible that the threshold could be affected by PM_10_ concentration. Temperature may also modify the effect of O_3_ on mortality. Ren et al. have reported different effect modifications by temperature according to the geographical location [36]. Nevertheless, the possible associations between threshold and other environmental factors should be considered as a hypothesis for further analysis, since we only used 7 data points and no statistical test was conducted.

We observed thresholds in 7 out of 13 cities. Although we chose metropolitan cities to secure power, some of the cities may still lack power. For instance, Daejon, Gwangju and Ulsan are cities with the smallest population, which showed a linear C-R relationship. The rest of the cities, Incheon, Osaka and Fukuoka satisfied only the criteria for non-linear C-R relationship without showing thresholds. Since the curves in these cities only showed downward slope in the lower concentration, the thresholds may still exist.

Stylianou et al. reported that they had observed linear C-R relationship for respiratory mortality, while reporting a non-linear relationship for cardiovascular mortality [34]. The result of the present study was consistent with that of the previous study. The mechanism of action of O_3_ exposure on cardiovascular system are still limited [33], but the mediators generated by inflammation and oxidative stress secondary to the direct irritation from exposure are thought to be the more likely cause of cardiovascular effect [8]. This mechanism may have caused non-linear C-R relationship between O_3_ concentration and cardiovascular mortality.

The city-combined models also had negative ΔAIC and J-shaped C-R relationship, even with adjustment for PM_10_ concentration. It was also considered to have a threshold between 30–40 ppb. The threshold was comparable to those of previous reports [17,18] and the background concentration of O_3_.

The present study also has limitations. Long-term exposure to O_3_ has been reported to cause adverse health effects [37]. However, the present study analyzed C-R relationship of acute exposure, which is inapplicable to the long-term effect of O_3_. The exposure was measured at the monitoring stations and this may cause exposure misclassification in the individual level. However, the measurement from monitoring stations reflected the daily variation relevant to the time-series analysis [1]. The exposure misclassification may also have attenuated the non-linearity of the C-R relationship [38], making it harder to detect. We presented the results of the present analyses using daily mean O_3_ concentration, but the sensitivity analysis using daily maximum 1-hour concentration also showed a non-linear C-R relationship and threshold. Smith et al. had analyzed the C-R relationship of 8-hour mean concentration and reported that it may also be non-linear [17]. Lastly, we controlled the seasonality with additional indicator variable for season, and this is considered to be more crude way of controlling seasonal variability. We did so because of the concern that under-smoothing the time trend by applying higher degree of freedom would remove too much temporal variance and eventually mask the non-linearity of C-R relationship. We included potential time-varying confounders, such as PM_10_ and temperature in the model, but other factors that are not accounted for in the present study, such as NO_2_, might have affected the results. The correlation between NO_2_ and O_3_ has been reported and the negative association below the threshold might be the result of this [39].

## Conclusion

There has been inconsistency of results regarding the shape of C-R relationship and existence of threshold in the association between ambient O_3_ and mortality. Our study provides an evidence supporting non-linearity and existence of a threshold. Until now, non-linearity was not accounted in the establishing of air quality standards; however the present study suggests that the non-linear C-R relationship and threshold should be considered in the future air quality standard. Our results also suggest that the threshold may vary according to other environmental factors, such as temperature and particulate matter concentration. This warrants further study exploring thresholds in different environmental settings.

## Supporting Information

S1 File Supplemental tables and figures. (PDF)

S1 Dataset Dataset used for the current analyses. (CSV)
