# Supplementary material for: Non-Linear Concentration-Response Relationships between Ambient Ozone and Daily Mortality
Source: PLoS One. 2015 Jun 15;10(6):e0129423. doi: 10.1371/journal.pone.0129423 (PMC4468145; doi:10.1371/journal.pone.0129423)
Supplement: S1 File — (PDF) [file pone.0129423.s001.pdf]

Supplemental Material

Title: Non-Linear Concentration-Response Relationships between Ambient Ozone and Daily Mortality

Authors: Sanghyuk Bae[1)], Youn-Hee Lim[2)], Saori Kashima[3)], Takashi Yorifuji[4)], Yasushi Honda[5)], Ho Kim[6)], Yun-Chul Hong[1)7)]

Affiliations: [1)] Department of Preventive Medicine, College of Medicine, Seoul National University, Seoul, Korea

[2)] Institutes of Health and Environment, Seoul National University, Seoul, Korea

[3)] Department of Public Health and Health Policy, Institute of Biomedical & Health Science, Hiroshima University, Hiroshima, Japan

[4)] Department of Human Ecology, Okayama University Graduate School of Environmental and Life Science, Okayama, Japan

[5)] Department of Health Care Policy and Health Economics, Faculty of Medicine, University of Tsukuba, Tsukuba, Japan

[6)] Department of Epidemiology and Biostatistics, Graduate School of Public Health, Seoul National University, Seoul, Korea

[7)] Institute of Environmental Medicine, Seoul National University Medical Research Center, Seoul, Korea

Table S1. Associations between daily mean O_3 and daily mortality in 13 Japanese and Korean cities from 2000 to 2009

City	Lag	Linear Model				Spline Model		ΔAIC
		Beta	SE	P	AIC	P	AIC	
Seoul	0	-0.000228	0.000191	0.2333	28000.5	0.0222	27992.5	-8.0
	1	-0.000372	0.000170	0.0288	27976.7	0.0002	27959.9	-16.7
	2	-0.000270	0.000163	0.0979	27955.3	0.0615	27951.8	-3.5
	3	-0.000381	0.000163	0.0190	27937.2	0.0176	27932.8	-4.4
	4	-0.000500	0.000163	0.0022	27908.4	0.0060	27906.0	-2.4
	5	-0.000440	0.000164	0.0073	27887.7	0.0073	27887.7	0.0
	0-1	-0.000441	0.000217	0.0422	27977.2	<0.0001	27929.4	-47.8
	0-2	-0.000500	0.000228	0.0282	27953.2	<0.0001	27916.7	-36.5
	0-3	-0.000603	0.000237	0.0109	27936.1	0.0002	27921.0	-15.1
	0-4	-0.000728	0.000246	0.0031	27908.8	0.0002	27897.4	-11.4
	0-5	-0.000820	0.000254	0.0012	27884.5	0.0009	27877.8	-6.7
Incheon	0	-0.000695	0.000315	0.0276	22748.7	0.0276	22748.7	0.0
	1	-0.000368	0.000303	0.2246	22747.5	0.2852	22747.0	-0.5
	2	-0.000002	0.000297	0.9940	22742.5	0.9943	22742.5	0.0
	3	-0.000006	0.000297	0.8371	22736.5	0.8371	22736.5	0.0
	4	-0.000292	0.000299	0.3285	22731.2	0.3385	22731.2	-0.1
	5	-0.000877	0.000300	0.0034	22718.8	0.0226	22717.9	-0.9
	0-1	-0.000721	0.000363	0.0473	22734.2	0.1221	22733.8	-0.4
	0-2	-0.000548	0.000390	0.1593	22718.8	0.1497	22716.2	-2.6
	0-3	-0.000484	0.000410	0.2372	22701.8	0.1030	22698.4	-3.4
	0-4	-0.000537	0.000427	0.2085	22683.6	0.1795	22681.8	-1.8
	0-5	-0.000801	0.000443	0.0706	22664.5	0.1578	22663.6	-0.9

Daejon	0	-0.000790	0.000486	0.1040	20118.9	0.1286	20118.6	-0.4
	1	-0.000935	0.000427	0.0287	20111.6	0.0892	20111.5	-0.1
	2	-0.000790	0.000409	0.0536	20108.1	0.0541	20108.1	0.0
	3	-0.000354	0.000406	0.3830	20088.2	0.3855	20088.2	0.0
	4	-0.000453	0.000405	0.2640	20090.9	0.4022	20091.0	0.1
	5	-0.000785	0.000405	0.0525	20080.0	0.0510	20077.4	-2.6
	0-1	-0.001175	0.000528	0.0261	20079.5	0.0262	20079.5	0.0
	0-2	-0.001301	0.000545	0.0170	20043.1	0.1559	20043.8	0.8
	0-3	-0.001186	0.000559	0.0338	19990.1	0.2030	19990.4	0.3
	0-4	-0.001210	0.000572	0.0344	19960.8	0.1117	19959.0	-1.8
	0-5	-0.001330	0.000583	0.0225	19925.1	0.0472	19921.6	-3.5
								0.0
Daegu	0	0.000319	0.000352	0.3648	22925.6	0.0535	22919.2	-6.4
	1	0.000035	0.000293	0.9057	22918.7	0.5760	22918.1	-0.7
	2	0.000181	0.000278	0.5154	22912.6	0.5155	22912.6	0.0
	3	-0.000479	0.000276	0.0821	22906.7	0.1286	22904.7	-2.0
	4	0.000138	0.000274	0.6157	22904.5	0.6195	22904.5	0.0
	5	-0.000352	0.000275	0.2007	22906.3	0.2019	22906.3	0.0
	0-1	0.000209	0.000376	0.5794	22906.1	0.1240	22901.7	-4.5
	0-2	0.000266	0.000384	0.4887	22888.9	0.2350	22886.1	-2.8
	0-3	-0.000040	0.000394	0.9197	22872.9	0.3797	22870.7	-2.2
	0-4	0.000006	0.000403	0.9889	22855.6	0.1951	22852.6	-3.0
	0-5	-0.000153	0.000411	0.7104	22835.7	0.4653	22834.8	-0.9
Gwangju	0	0.000614	0.000498	0.2170	20287.9	0.2770	20286.4	-1.5
	1	-0.000059	0.000460	0.8990	20286.3	0.8990	20286.3	0.0
	2	0.000326	0.000445	0.4640	20283.6	0.4650	20283.6	0.0

	3	0.000426	0.000442	0.3350	20273.7	0.3280	20272.9	-0.8
	4	0.000128	0.000443	0.7730	20263.7	0.7730	20263.7	0.0
	5	-0.000210	0.000443	0.6360	20260.0	0.6370	20260.0	0.0
	0-1	0.000333	0.000551	0.5460	20267.8	0.5310	20267.9	0.1
	0-2	0.000450	0.000577	0.4360	20251.3	0.1250	20246.7	-4.6
	0-3	0.000567	0.000596	0.3420	20231.8	0.0881	20226.4	-5.4
	0-4	0.000534	0.000613	0.3840	20211.9	0.0363	20203.7	-8.2
	0-5	0.000446	0.000628	0.4780	20191.3	0.0559	20184.0	-7.4
Busan	0	0.000104	0.000266	0.6960	24853.0	0.0230	24841.1	-11.9
	1	0.000054	0.000243	0.8240	24836.1	0.4746	24835.2	-1.0
	2	0.000413	0.000240	0.3326	24827.3	0.0851	24827.3	0.0
	3	0.000308	0.000239	0.1980	24817.2	0.1980	24817.2	0.0
	4	-0.000011	0.000240	0.9649	24802.1	0.7566	24801.9	-0.2
	5	0.000001	0.000240	0.9974	24787.3	0.9971	24787.3	0.0
	0-1	0.000107	0.000294	0.7161	24829.5	0.0026	24814.6	-14.9
	0-2	0.000322	0.000310	0.2997	24813.4	0.0208	24804.8	-8.6
	0-3	0.000428	0.000325	0.1882	24797.5	0.0731	24792.9	-4.6
	0-4	0.000383	0.000339	0.2585	24775.6	0.0374	24769.2	-6.5
	0-5	0.000375	0.000350	0.2849	24755.1	0.1311	24751.4	-3.7
Ulsan	0	0.000189	0.000629	0.7640	18745.5	0.1272	18740.3	-5.2
	1	-0.000359	0.000561	0.5220	18745.7	0.5221	18745.7	0.0
	2	-0.000313	0.000542	0.5630	18731.6	0.5647	18731.6	0.0
	3	-0.000065	0.000541	0.9040	18727.1	0.7267	18726.8	-0.3
	4	0.000106	0.000542	0.8450	18720.6	0.4949	18719.8	-0.8
	5	-0.000629	0.000545	0.2480	18708.5	0.2486	18708.5	0.0
	0-1	-0.000143	0.000690	0.8360	18711.0	0.8361	18711.0	0.0

	0-2	-0.000285	0.000721	0.6930	18668.4	0.6933	18668.4	0.0
	0-3	-0.000267	0.000751	0.7220	18634.1	0.5995	18634.5	0.4
	0-4	-0.000216	0.000778	0.7810	18602.1	0.3772	18600.3	-1.8
	0-5	-0.000384	0.000804	0.6330	18560.7	0.5731	18561.2	0.5
Sapporo	0	-0.000041	0.000312	0.8961	23369.3	0.8270	23369.3	-0.1
	1	-0.000448	0.000305	0.1417	23361.8	0.1420	23361.8	0.0
	2	-0.000346	0.000301	0.2499	23357.4	0.2500	23357.4	0.0
	3	-0.000235	0.000301	0.4343	23347.9	0.4350	23347.9	0.0
	4	-0.000227	0.000302	0.4527	23344.5	0.4530	23344.5	0.0
	5	-0.000212	0.000303	0.4847	23336.2	0.0777	23330.2	-6.1
	0-1	-0.000290	0.000333	0.3851	23358.0	0.3850	23358.0	0.0
	0-2	-0.000359	0.000344	0.2959	23346.8	0.2960	23346.8	0.0
	0-3	-0.000351	0.000351	0.3175	23331.6	0.3180	23331.6	0.0
	0-4	-0.000360	0.000357	0.3132	23319.0	0.3130	23319.0	0.0
	0-5	-0.000348	0.000362	0.3364	23305.2	0.3370	23305.2	0.0
Tokyo	0	-0.000585	0.000130	<0.0001	30606.4	<0.0001	30584.8	-21.6
	1	-0.000808	0.000117	<0.0001	30570.0	<0.0001	30546.0	-24.0
	2	-0.000324	0.000115	0.0049	30602.8	0.0180	30601.2	-1.6
	3	-0.000374	0.000116	0.0012	30591.2	0.0012	30591.2	0.0
	4	-0.000626	0.000116	<0.0001	30565.5	<0.0001	30565.5	0.0
	5	-0.000564	0.000117	<0.0001	30563.7	<0.0001	30557.5	-6.2
	0-1	-0.000893	0.000138	<0.0001	30575.8	<0.0001	30543.4	-32.4
	0-2	-0.000810	0.000143	<0.0001	30578.5	<0.0001	30556.2	-22.4
	0-3	-0.000803	0.000148	<0.0001	30572.1	<0.0001	30557.0	-15.1
	0-4	-0.000897	0.000153	<0.0001	30559.8	<0.0001	30550.2	-9.5
	0-5	-0.000956	0.000157	<0.0001	30549.5	<0.0001	30543.6	-5.9

Nagoya	0	-0.000140	0.000290	0.6279	24752.4	0.3470	24750.2	-2.1
	1	-0.000571	0.000254	0.0246	24742.0	0.0051	24734.8	-7.2
	2	0.000038	0.000240	0.8755	24741.1	0.8758	24741.1	0.0
	3	0.000356	0.000242	0.1403	24729.1	0.1405	24729.1	0.0
	4	0.000038	0.000241	0.8748	24725.7	0.1434	24720.9	-4.7
	5	-0.000376	0.000242	0.1210	24717.4	0.0653	24712.5	-4.9
	0-1	-0.000499	0.000308	0.1053	24744.4	0.0248	24737.8	-6.6
	0-2	-0.000321	0.000313	0.3050	24740.2	0.0458	24734.1	-6.1
	0-3	-0.000090	0.000320	0.7788	24731.3	0.1413	24727.2	-4.0
	0-4	-0.000061	0.000327	0.8517	24725.6	0.1424	24721.3	-4.4
	0-5	-0.000174	0.000334	0.6019	24719.6	0.2248	24716.5	-3.1
Osaka	0	-0.000239	0.000223	0.2838	26441.9	0.2840	26441.9	0.0
	1	-0.000745	0.000195	0.0001	26421.1	0.0001	26413.7	-7.4
	2	0.000143	0.000183	0.4336	26429.2	0.4342	26429.2	0.0
	3	0.000019	0.000183	0.9158	26424.5	0.5989	26423.7	-0.8
	4	-0.000160	0.000183	0.3810	26413.3	0.3811	26413.3	0.0
	5	0.000161	0.000184	0.3801	26406.5	0.3802	26406.5	0.0
	0-1	-0.000741	0.000247	0.0027	26426.6	0.0052	26424.5	-2.2
	0-2	-0.000436	0.000255	0.0871	26426.8	0.1453	26425.7	-1.1
	0-3	-0.000337	0.000264	0.2016	26422.9	0.2019	26422.9	0.0
	0-4	-0.000363	0.000273	0.1837	26412.3	0.1836	26412.3	0.0
	0-5	-0.000261	0.000282	0.3553	26406.4	0.3552	26406.4	0.0
Kitakyushu	0	0.000067	0.000327	0.8387	22370.9	0.8397	22370.9	0.0
	1	0.000474	0.000310	0.1264	22362.6	0.0278	22356.0	-6.6
	2	0.000500	0.000291	0.0857	22354.1	0.1741	22353.8	-0.4

	3	0.001051	0.000290	0.0003	22334.0	0.0003	22334.0	0.0
	4	0.000486	0.000291	0.0945	22339.0	0.0946	22339.0	0.0
	5	0.000403	0.000291	0.1669	22334.6	0.1690	22334.6	0.0
	0-1	0.000399	0.000374	0.2859	22363.8	0.1017	22359.8	-4.0
	0-2	0.000616	0.000392	0.1161	22354.5	0.0762	22351.9	-2.6
	0-3	0.001008	0.000405	0.0128	22341.0	0.0210	22339.5	-1.5
	0-4	0.001062	0.000418	0.0111	22335.2	0.0113	22335.2	0.0
	0-5	0.001083	0.000429	0.0117	22329.9	0.0117	22329.9	0.0
Fukuoka	0	-0.000140	0.000354	0.6929	21993.0	0.6940	21993.0	0.0
	1	-0.000465	0.000340	0.1712	21985.1	0.1720	21985.1	0.0
	2	-0.000031	0.000318	0.9212	21981.8	0.9220	21981.8	0.0
	3	-0.000120	0.000316	0.7046	21976.1	0.7060	21976.1	0.0
	4	-0.000053	0.000316	0.8671	21969.8	0.6460	21968.9	-0.9
	5	-0.000485	0.000317	0.1262	21961.6	0.1260	21961.6	0.0
	0-1	-0.000405	0.000397	0.3080	21985.9	0.0306	21977.1	-8.9
	0-2	-0.000316	0.000415	0.4466	21981.1	0.2460	21978.6	-2.5
	0-3	-0.000310	0.000427	0.4676	21975.7	0.4010	21974.8	-0.8
	0-4	-0.000280	0.000439	0.5238	21969.3	0.5240	21969.3	0.0
	0-5	-0.000404	0.000449	0.3686	21963.2	0.3690	21963.2	0.0

ΔAIC=AIC of Spline Model – AIC of Linear Model

Table S2. Associations between daily mean O3(lag0-1) and daily number of mortality adjusting for different temperature lags in 13 Japanese and Korean cities from 2000 to 2009

City	Temp. Lag	Linear Model				Spline Model		ΔAIC
		Beta	SE	P	AIC	P	AIC	
Seoul	0	-0.00003	0.000230	0.8945	27968.5	0.0000	27920.0	-48.5
	0-1	0.00007	0.000231	0.7749	27943.6	0.0001	27925.0	-18.6
	0-3	0.00017	0.000229	0.4686	27880.5	0.0001	27862.7	-17.8
	0-7	0.00023	0.000224	0.3124	27776.0	0.0009	27763.8	-12.2
	0-14	0.00015	0.000223	0.5072	27691.2	0.0034	27681.1	-10.1
	0-28	0.00011	0.000222	0.6263	27625.6	0.0193	27618.7	-6.9
Incheon	0	-0.00088	0.000388	0.1356	22737.8	0.1799	22737.1	-0.7
	0-1	-0.00057	0.000388	0.1434	22729.4	0.2129	22728.9	-0.6
	0-3	-0.00057	0.000390	0.1421	22705.6	0.3080	22705.3	-0.2
	0-7	-0.00063	0.000393	0.1108	22679.0	0.1798	22678.9	0.0
	0-14	-0.00059	0.000395	0.1371	22634.6	0.1917	22637.2	2.6
	0-28	-0.00058	0.000393	0.1380	22561.2	0.1381	22561.2	0.0
Daejon	0	-0.00024	0.000542	0.6620	20056.7	0.6633	20056.7	0.0
	0-1	-0.00019	0.000547	0.7220	20054.2	0.7223	20054.2	0.0
	0-3	-0.00019	0.000548	0.7326	20044.7	0.7329	20044.7	0.0
	0-7	-0.00027	0.000546	0.6257	20017.2	0.6258	20017.2	0.0
	0-14	-0.00037	0.000553	0.4991	19976.2	0.5000	19976.2	0.0
	0-28	-0.00046	0.000544	0.4006	19893.0	0.4012	19893.0	0.0

Daegu	0	0.00063	0.000384	0.1019	22898.4	0.0426	22894.1	-4.3
	0-1	0.00073	0.000385	0.0570	22879.5	0.0316	22875.5	-4.0
	0-3	0.00059	0.000384	0.1269	22855.4	0.0948	22852.9	-2.5
	0-7	0.00047	0.000385	0.2177	22818.2	0.3336	22817.7	-0.5
	0-14	0.00041	0.000383	0.2866	22771.5	0.5396	22771.4	-0.1
	0-28	0.00036	0.000384	0.3493	22697.5	0.3390	22697.2	-0.3
Gwangju	0	0.00103	0.000570	0.0698	20247.6	0.0698	20247.6	0.0
	0-1	0.00106	0.000571	0.0629	20243.6	0.0630	20243.6	0.0
	0-3	0.00093	0.000567	0.1018	20233.2	0.1019	20233.2	0.0
	0-7	0.00080	0.000540	0.1373	20197.1	0.1443	20197.1	0.0
	0-14	0.00083	0.000538	0.1226	20140.5	0.1487	20140.3	-0.3
	0-28	0.00075	0.000535	0.1629	20058.5	0.2593	20057.9	-0.7
Busan	0	0.00091	0.000310	0.0035	24783.3	0.0000	24760.5	-22.8
	0-1	0.00096	0.000312	0.0021	24770.4	0.0000	24747.9	-22.5
	0-3	0.00096	0.000312	0.0020	24730.6	0.0000	24711.9	-18.7
	0-7	0.00104	0.000307	0.0007	24670.2	0.0000	24655.7	-14.4
	0-14	0.00106	0.000300	0.0004	24608.9	0.0000	24596.8	-12.1
	0-28	0.00125	0.000294	0.0000	24497.0	0.0000	24490.2	-6.9
Ulsan	0	0.00092	0.000659	0.1626	18692.2	0.1627	18692.2	0.0
	0-1	0.00097	0.000659	0.1400	18690.9	0.1400	18690.9	0.0
	0-3	0.00081	0.000692	0.2439	18675.7	0.2440	18675.7	0.0
	0-7	0.00100	0.000651	0.1252	18646.1	0.1254	18646.1	0.0

	0-14	0.00083	0.000672	0.2159	18603.6	0.2161	18603.6	0.0
	0-28	0.00071	0.000666	0.2830	18526.6	0.2832	18526.6	0.0
Sapporo	0	0.00085	0.000387	0.0277	23342.2	0.0479	23341.2	-1.0
	0-1	0.00082	0.000389	0.0362	23330.1	0.0675	23329.4	-0.7
	0-3	0.00064	0.000400	0.1094	23297.8	0.2218	23297.3	-0.4
	0-7	0.00039	0.000382	0.3130	23250.4	0.5204	23250.2	-0.1
	0-14	0.00015	0.000381	0.7025	23149.6	0.7026	23149.6	0.0
	0-28	-0.00001	0.000397	0.9784	22955.4	0.9853	22955.4	0.0
Tokyo	0	-0.00013	0.000160	0.4112	30555.9	0.0000	30530.0	-25.8
	0-1	0.00004	0.000159	0.7796	30402.0	0.0003	30382.3	-19.7
	0-3	0.00011	0.000160	0.4912	30169.1	0.0002	30149.0	-20.2
	0-7	-0.00010	0.000162	0.5353	30022.7	0.0002	30001.8	-21.0
	0-14	-0.00035	0.000163	0.0294	30065.1	0.0000	30043.7	-21.4
	0-28	-0.00081	0.000161	0.0000	30096.6	0.0000	30058.9	-37.7
Nagoya	0	0.00018	0.000352	0.6140	24744.9	0.0558	24738.2	-6.7
	0-1	0.00025	0.000350	0.4779	24687.4	0.0949	24682.7	-4.7
	0-3	0.00023	0.000351	0.5126	24586.2	0.2168	24583.5	-2.7
	0-7	0.00007	0.000354	0.8385	24491.0	0.4395	24489.9	-1.2
	0-14	-0.00005	0.000359	0.8813	24399.9	0.4006	24398.3	-1.6
	0-28	-0.00021	0.000362	0.5528	24231.5	0.3362	24229.1	-2.4
Osaka	0	-0.00070	0.000272	0.0103	26459.5	0.0165	26457.6	-1.9

	0-1	-0.00053	0.000272	0.0494	26374.7	0.0564	26374.5	-0.2
	0-3	-0.00033	0.000271	0.2287	26234.8	0.2287	26234.8	0.0
	0-7	-0.00026	0.000271	0.3468	26094.7	0.3469	26094.7	0.0
	0-14	-0.00039	0.000271	0.1497	25910.0	0.1497	25910.0	0.0
	0-28	-0.00072	0.000270	0.0080	25562.0	0.0080	25562.0	0.0
Kitakyushu	0	0.00057	0.000414	0.1686	22369.5	0.1060	22365.5	-4.0
	0-1	0.00080	0.000417	0.0538	22324.0	0.1219	22322.7	-1.3
	0-3	0.00071	0.000413	0.0850	22245.2	0.0892	22245.2	0.0
	0-7	0.00055	0.000409	0.1745	22195.4	0.1749	22195.4	0.0
	0-14	0.00036	0.000405	0.3785	22151.9	0.3786	22151.9	0.0
	0-28	0.00005	0.000395	0.8939	22073.6	0.8938	22073.6	0.0
Fukuoka	0	-0.00017	0.000445	0.7108	21991.2	0.02583	21981.3	-10.0
	0-1	-0.00017	0.000443	0.7088	21978.8	0.04120	21970.2	-8.6
	0-3	-0.00033	0.000435	0.4435	21947.7	0.04373	21939.6	-8.1
	0-7	-0.00046	0.000421	0.2727	21909.2	0.03635	21901.1	-8.0
	0-14	-0.00076	0.000411	0.0657	21869.1	0.01677	21861.6	-7.5
	0-28	-0.00112	0.000398	0.0050	21802.0	0.00281	21794.0	-8.0

ΔAIC=AIC of Spline Model – AIC of Linear Model

Table S3. Associations between daily mean O_3 (lag_{0-1}, ppb) and daily number of deaths from cardiovascular and respiratory diseases in 13 Japanese and Korean cities from 2000 to 2009

Cause of Death	City	Linear Model				Spline Model		ΔAIC
		Beta	SE	P	AIC	P	AIC	
Cardiovascular	Seoul	-0.000827	0.000442	0.0615	22283.9	<0.0001	22251.9	-32.0
	Incheon	-0.000689	0.000717	0.3364	17617.3	0.3370	17617.3	0.0
	Daejon	-0.002485	0.001068	0.0200	14740.7	0.0201	14740.7	0.0
	Daegu	0.000545	0.000756	0.4710	17433.8	0.4790	17433.8	0.0
	Gwangju	0.000778	0.001214	0.5210	14339.4	0.2940	14336.7	-2.6
	Busan	-0.000448	0.000549	0.4142	19901.6	0.4830	19900.9	-0.7
	Ulsan	0.002903	0.001295	0.0250	13334.3	0.0794	13333.5	-0.8
	Sapporo	-0.000006	0.000606	0.9921	18849.8	0.6230	18849.1	-0.6
	Tokyo	-0.001169	0.000253	<0.0001	25415.2	<0.0001	25387.6	-27.6
	Nagoya	-0.001024	0.000567	0.0706	20165.7	0.0093	20158.3	-7.4
	Osaka	-0.000131	0.000473	0.7819	21365.6	0.7820	21365.6	0.0
	Kitakyushu	0.001255	0.000698	0.0721	17405.9	0.1302	17405.9	0.0
	Fukuoka	-0.000896	0.000790	0.2568	16746.2	0.0549	16740.8	-5.4
Respiratory	Seoul	0.002757	0.000943	0.0035	16538.4	0.0035	16538.4	0.0
	Incheon	0.001919	0.001496	0.1994	11719.3	0.2000	11719.3	0.0
	Daejon	0.001526	0.001962	0.4367	9510.0	0.1153	9505.9	-4.1
	Daegu	0.005671	0.001515	0.0002	11972.3	0.0007	11970.5	-1.8
	Gwangju	-0.000025	0.002143	0.9910	9082.5	0.1870	9079.0	-3.5
	Busan	0.002247	0.001202	0.0616	13754.4	0.0201	13747.4	-7.0
	Ulsan	0.003623	0.002554	0.1560	7918.7	0.1561	7918.7	0.0
	Sapporo	0.001289	0.000922	0.1620	15627.1	0.2910	15627.1	-0.1

Tokyo	-0.000448	0.000368	0.2230	22550.3	0.3696	22550.2	0.0
Nagoya	0.001085	0.000819	0.1852	17096.1	0.1860	17096.1	0.0
Osaka	0.000614	0.000629	0.3290	18966.4	0.3290	18966.4	0.0
Kitakyushu	0.001909	0.000960	0.0468	14993.1	0.0533	14989.7	-3.4
Fukuoka	-0.000357	0.000921	0.6984	14770.5	0.8520	14771.4	0.9

ΔAIC=AIC of Spline Model – AIC of Linear Model

Table S4. Associations between daily maximum 1-hour O_3 (ppb) and daily mortality in Korean cities from 2000 to 2009

City	Linear Model				Spline Model		ΔAIC
	Beta	SE	P	AIC	P	AIC	
Seoul	0.000011	0.000127	0.9297	28035.0	0.0072	28025.6	-9.4
Incheon	-0.000505	0.000225	0.0246	22754.4	0.0248	22754.4	0.0
Daejon	0.000191	0.000355	0.5900	20119.4	0.2321	20115.7	-3.7
Daegu	0.000454	0.000243	0.0618	22921.8	0.0875	22918.4	-3.4
Gwangju	0.000641	0.000360	0.0754	20287.2	0.0980	20287.0	-0.2
Busan	0.000464	0.000200	0.0204	24845.3	0.0016	24833.8	-11.4
Ulsan	0.000223	0.000447	0.6180	18744.5	0.3540	18742.6	-1.9

ΔAIC=AIC of Spline Model – AIC of Linear Model

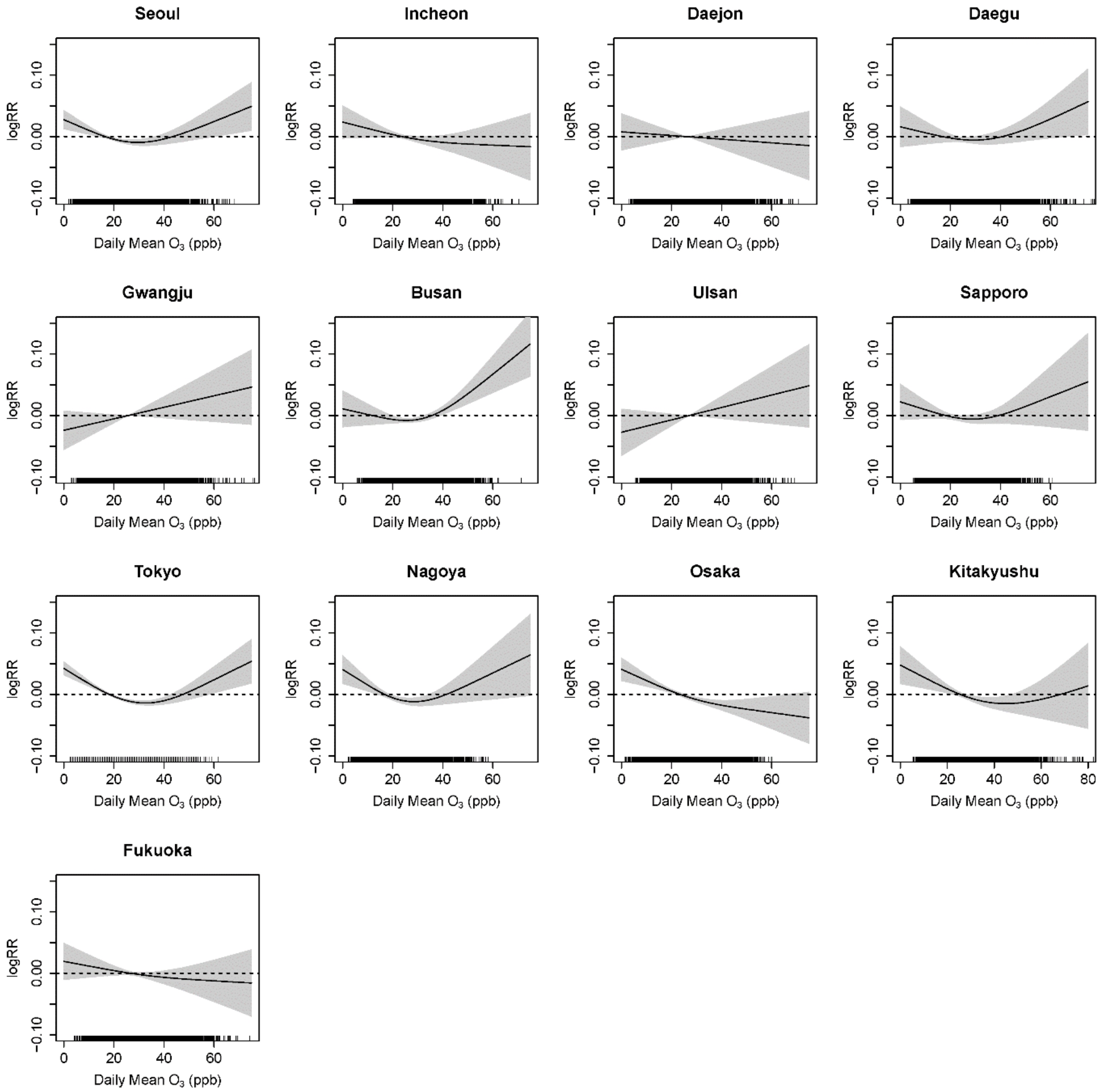

Figure S1. Associations between daily mean O_3 (lag0-1, ppb) and daily mortality adjusting for PM_{10} in 13 Japanese and Korean cities from 2000 to 2009

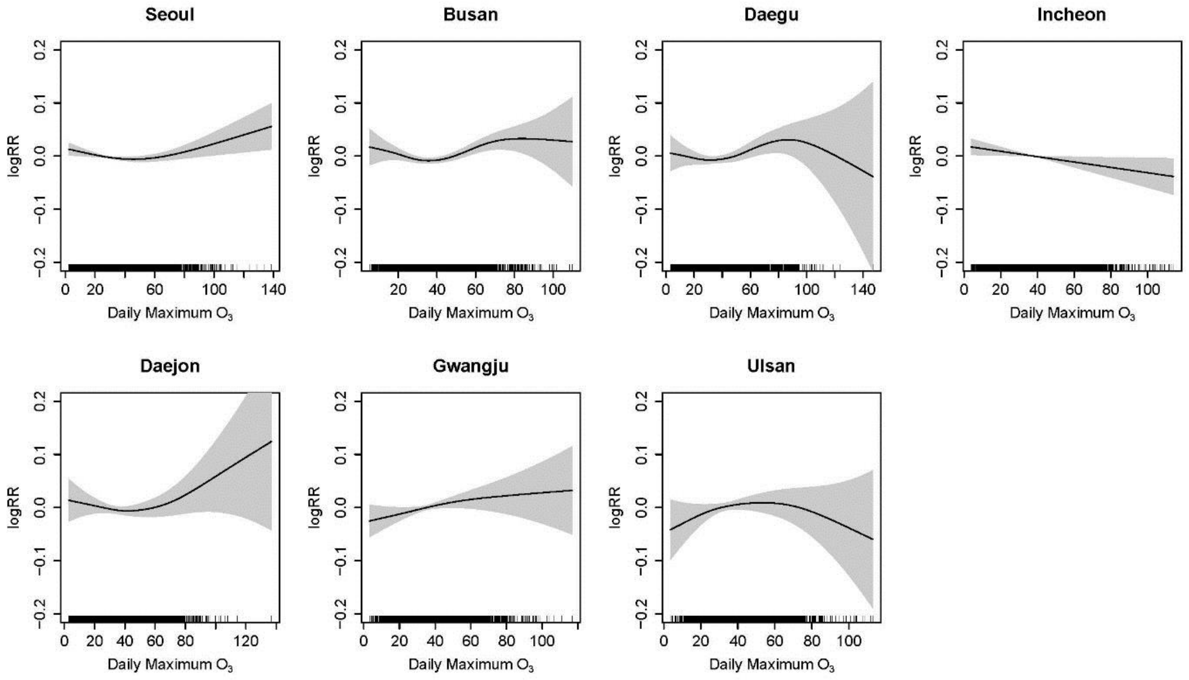

Figure S2. Associations between daily maximum 1-hour O_3 (ppb) concentration and daily mortality in 7 Korean cities from 2000 to 2009

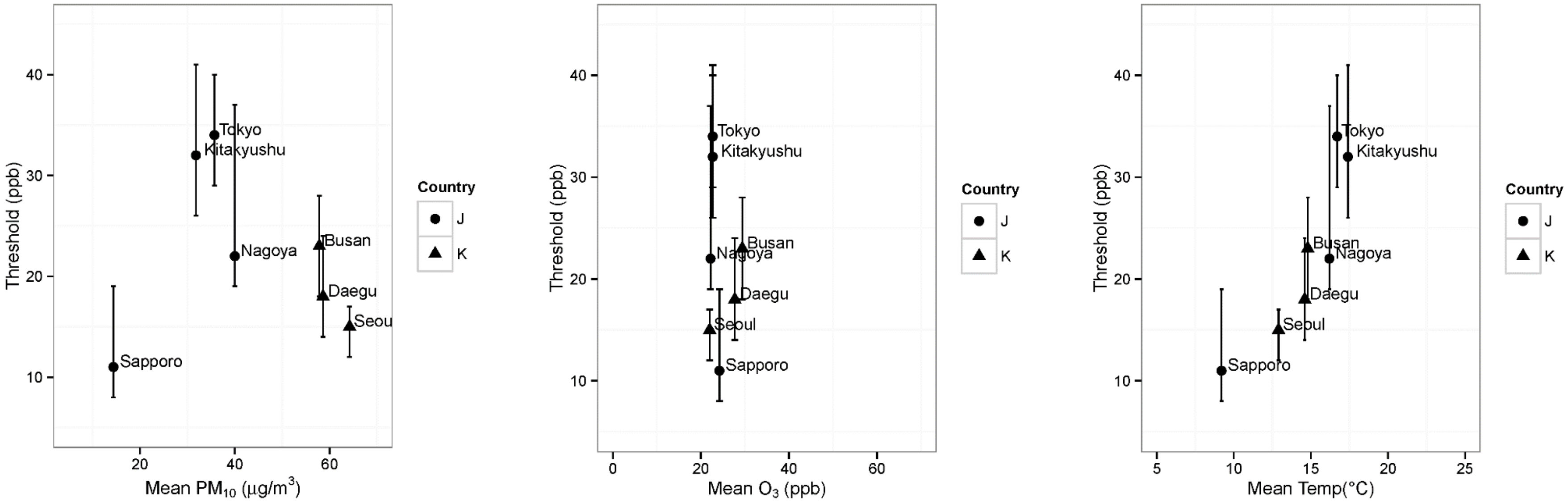

Fig. S3. Selected city-specific factors and the thresholds of concentration-response relationship between O_3 concentration and daily mortality in the 7 cities with non-linear association

J: Japan, K: Korea
